# Underweight and risk of fractures in adults over 40 years using the nationwide claims database

**DOI:** 10.1038/s41598-023-34828-y

**Published:** 2023-05-17

**Authors:** Sang-Min Park, Jiwon Park, Sangsoo Han, Hae-Dong Jang, Jae-Young Hong, Kyungdo Han, Ho-Joong Kim, Jin S. Yeom

**Affiliations:** 1 Spine Center and Department of Orthopaedic Surgery, Seoul National University College of Medicine and Seoul National University Bundang Hospital, Seongnam, Korea; 2 Department of Orthopaedic Surgery, Korea University Ansan Hospital, Ansan, 123, Jeokgeum-Ro, Danwon-Gu, Ansan-Si, Gyeonggi-Do, 15355 Republic of Korea; 3 Department of Emergency Medicine, Soonchunhyang University Bucheon Hospital, 170 Jomaru-Ro, Bucheon, 14584 Republic of Korea; 4 Department of Orthopaedic Surgery, Soonchunhyang University Bucheon Hospital, 170 Jomaru-Ro, Bucheon, 14584 Republic of Korea; 5 Department of Statistics and Actuarial Science, Soongsil University, 369 Sangdo-Ro, Dongjak-Gu, Seoul, 06978 Republic of Korea

**Keywords:** Trauma, Bone

## Abstract

We aimed to investigate how underweight affects the incidence of fractures, as well as the influence of cumulative, longitudinal periods of low body mass index (BMI) and changes in body weight on fracture development. Data on adults aged 40-year and over who had three health screenings between January 1, 2007, and December 31, 2009 were used to determine the incidence of new fractures. The hazard ratios (HRs) for new fractures depending on BMI, total cumulative number of underweight, and weight change over time were calculated using Cox proportional hazard analysis. In this study, 15,955 (2.8%) of the 561,779 adults were diagnosed with fractures more than once over three health examinations. The fully adjusted HR for fractures in underweight individuals was 1.173 (95% Confidence interval [CI] 1.093–1.259). Underweight individuals diagnosed only once, twice, or three times had an adjusted HR of 1.227 (95%CI 1.130–1.332), 1.174 (95%CI 1.045–1.319), and 1.255 (95%CI 1.143–1.379), respectively. Although the adjusted HR was higher in adults who consistently had underweight (HR; 1.250 [95%CI 1.146–1.363]), those with underweight had an increased risk of fractures regardless of weight change (HR; 1.171 [95%CI 1.045–1.312], and 1.203[95%CI 1.075–1.346]). Underweight is a risk factor for fractures in adults over the age of 40 years, even if they returned to normal weight.

## Introduction

Fractures are one of the leading causes of morbidity and mortality among adults, particularly the elderly^1^. Fractures are also directly related with increased social expenses, as they can result in extended absences, substantial use of medical resources, and long-term impairment^2^. Age, gender, menopause, underweight, obesity, smoking, excessive alcohol use, and lack of physical activity are well-known risk factors associated with an increase in fractures^3–11^. Weight loss has been linked to osteoporosis and sarcopenia, and weight gain has been demonstrated to help maintain bone density^12,13^. Therefore, it may be assumed that weight loss affects bone density and raises the risk of fractures, whereas, weight gain maintains bone density and reduces the incidence of fractures.

Weight is a major determinant of health status, including metabolic, immunological, reproductive, and musculoskeletal functioning^14^. Being underweight can result in poor physical health, which is directly related with an increased risk of mortality and morbidity^15,16^. Additionally, being underweight may be linked to reduced bone density, soft tissue loss, and muscle weakness; hence, increasing the risk of fractures^13^. However, weight gain without an increase in muscle mass did not prevent fractures, but rather increased their incidence^17^. Due to unfavorable attitudes and discrimination against obesity, a greater proportion of adults, particularly women, are underweight in modern culture^18^. Therefore, it is crucial to examine the relationship between underweight and fractures. Consequently, we attempted to assess the risk of fracture resulting from an underweight using a database containing health examination results from the general Korean population. The purpose of this study was to investigate how being underweight affects the incidence of fractures, as well as the influence of cumulative numbers of low BMI on fracture development.

## Methods

### Data source, study design and population

The study protocol was approved by the Institutional Review Board of Korea University Ansan Hospital (Approval No. K2021-2601-001). The ethics committees of Korea University Ansan Hospital have waived the requirement to obtain informed consent as the register data analysed in this study are in anonymised and deidentified format. This study was performed in accordance with the tenets of the Declaration of Helsinki, and all research methods were carried out in accordance with appropriate regulations and guidelines.

The KNHIS database contains health information of the entire Korean population (approximately 50 million people), including diagnoses (ICD-10) and prescriptions as well as procedures^19^. All insured Koreans aged 40 years and older and all workers aged 20 years and older must undergo regular health screening examinations one or two years^20^. Among the information contained in these health-screening records include anthropometric measurements and lifestyle questionnaires, socioeconomic data and records of prescriptions and hospitalizations as well as outpatient records and death dates for the insured Korean population.

**Figure 1:**
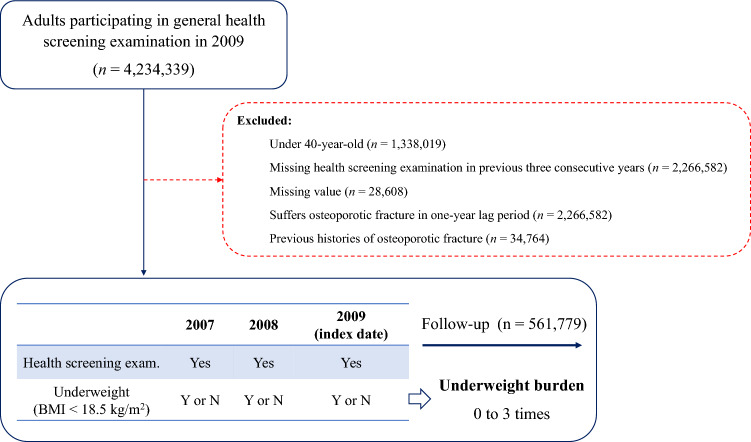
Flow chart of study population.

Data on adults over the age of 40 who had three consecutive general health tests between January 1, 2007, and December 31, 2009 was collected from this database and used to establish a long-term cohort study. Patients who previously suffered from osteoporotic fractures and with incomplete information were excluded from the study. The impact of being underweight was amplified by applying a one-year time lag after the screening process had been carried out. In total, this research included 561,779 participants (Fig. 1). Fracture cases were tracked in this cohort from the time of initial health assessment to the end of the cohort's designated follow-up period (December 2018) or the participant's death. Fractures were defined as any fracture that resulted in a claim for hospitalization or outpatient treatment after the index general health-screening date.

### Evaluation of body weight

This information was taken from the general health screening results. BMI was calculated as: weight in kilograms divided by their height in meters squared (kg/m^2^). Underweight (< 18.5), normal (≥ 18.5 and < 23), overweight (≥ 23 and < 25), obesity (≥ 25 and < 30), and severely obesity (≥ 30) were defined by the WHO Asia–Pacific regional guidelines^21,22^. The cumulative number of underweight diagnosed at each health screening examinations (0 to 3 times) was counted and divided into four groups.

Each time a patient was screened, their body weight status was reported. The total number of people who were identified to be overweight or obese as a result of routine health examinations was used to calculate the number of people who were actually underweight. As part of our study, we evaluated the diagnoses of underweight status at the first and final health screenings to see how BMI changes over time could affect fractures. There were four groups of people in the study: underweight to underweight (U-to-U), underweight to non-underweight (U-to-N), non-underweight to underweight (N-to-U), and non-underweight to non-underweight (N-to-N).

### Operational definitions of fractures

We utilized ICD-10, procedure, and radiographic study codes to search all the fracture cases from the insurance claim database^1,23,24^. ICD-10 codes for each fractures were the following: vertebral fracture [S22.0 (fracture of the thoracic spine), S22.1 (multiple fractures of the thoracic spine), S32.0 (fracture of the lumbar spine), S32.7 (multiple fractures of the lumbar spine), T080 (fracture of the spine), M48.4 (fatigue fracture of vertebra)], hip fracture [S72.0 (fracture of the femur neck) and S72.1 (trochanteric fracture)], humerus fracture [S42.2 (fracture of upper end of humerus), S42.3 (fractured shaft of humerus)], and radius fractures [S52.5 (fracture of lower end of radius) and S52.6 (fracture of lower end of both ulnar and radius)]^23^.

### Covariates and measurements

In this study, baseline demographic data were defined as those from the most recent health screening. Socioeconomic data, laboratory results (cholesterol, fasting glucose, blood pressure, triglyceride), responses to lifestyle questionnaires (regular exercise, smoking, alcohol consumption), anthropometric measurements (height, weight, waist circumference), and medical histories, which included hypertension, diabetes, dyslipidemia, and chronic kidney disease (CKD), comprised these fundamental characteristics^25^. Regarding medical history, comorbidities were provided if a record at the health screening or past medical claim data indicated their presence.

Non-smokers, former smokers, and current smokers were distinguished by their smoking status. According to the amount of alcohol consumed daily, participants were categorized as non-drinkers, light drinkers (less than 30 g/day), or heavy drinkers (more than 30 g/day). Regular exercise was defined as at least 20 min of vigorous exercise on at least three days per week or 30 min of moderate to intense exercise on at least five days per week. The income was classified as low if it fell within the bottom 20 percent of the yearly income, or as normal otherwise. Appendix I is a listing of the ICD-10 codes utilized for this investigation.

### Statistical analysis

According to the total number of underweight patients, baseline parameters of the study population are reported as mean (± standard deviation) or counts (percentages). The incidence rate (IR) per 1,000 person-years (PY) with 95% confidence intervals (95%CIs) was used to define the IR. We calculated the hazard ratios (HRs) with 95%CIs for the incidence of fractures by the BMI at the time of the index health screening examination (3rd exam; 2009) and the cumulative numbers of underweight using Cox’s regression analysis. The proportional-hazards assumption was assessed using the Schoenfeld residuals test, with a logarithm of the cumulative hazard functions based on Kaplan–Meier estimates^26^. Over time, there was no significant departure from proportionality in the hazards. To decrease covariate bias, we compared HRs for unadjusted and three adjusted models: Model 1 was adjusted for age and sex; Model 2 was adjusted for age, sex, and additional environmental factors including smoking, alcohol consumption, regular exercise, and low income; and Model 3 was fully adjusted for age, sex, additional environmental factors (smoking, alcohol consumption, regular exercise, and income), and comorbidities (diabetes, hypertension, dyslipidemia, and CKD). Statistical analysis was conducted with the SAS 9.3 program (SAS Institute, Cary, NC, USA). The analysis of variance for continuous variables and the chi-square test for categorical variables were utilized, and a two-sided p < 0.05 was regarded statistically significant.

## Results

### Baseline characteristics

Table 1 provides a summary of the baseline characteristics according to the cumulative number of underweight participants at each health screening examination. Of the total 561,779 participants, 545,824 (97.2%) had never been diagnosed as underweight. Regarding those who were underweight, 5,354 (1.0%) were diagnosed thrice, 3,672 (0.7%) were diagnosed twice, and 6,929 (1.2%) were diagnosed only once over the three health screenings. Except for age, the four groups of never-diagnosed, once-diagnosed, twice-diagnosed, and thrice-diagnosed individuals, indicated statistically significant differences in all categories investigated. Regardless of the duration of underweight status, those in the underweight group were more likely than those in the non-underweight group to be current smokers, to abstain from alcohol intake, to engage in regular exercise, and to have a low income.

**Table 1:** Baseline characteristics of this study according to the cumulative number of the presence of underweight.

Variables	Accumulated number of underweight* cases by health screening^†^				p-value
	0	1	2	3	
Participants (n)	545,824	6,929	3,672	5,354	
Age (years)	49.69 ± 7.14	49.73 ± 7.77	49.67 ± 7.78	49.58 ± 7.87	0.6816
Sex (n)					< .0001
Men	403,926(74)	4,270(61.63)	2,373(64.62)	3,495(65.28)	
Women	141,898(26)	2,659(38.37)	1,299(35.38)	1,859(34.72)	
Height (cm)	165.72 ± 8.03	163.99 ± 8.23	164.4 ± 7.9	164.81 ± 7.86	< .0001
Weight (kg)	66.39 ± 10.19	51.26 ± 6.2	49.62 ± 5.22	47.35 ± 4.99	< .0001
Smoking (n)					< .0001
Non	257,094(47.1)	3,699(53.38)	1,808(49.24)	2,683(50.11)	
Ex	127,920(23.44)	960(13.85)	515(14.03)	580(10.83)	
Current	160,810(29.46)	2,270(32.76)	1,349(36.74)	2,091(39.05)	
Alcohol consumption(n)^‡^					< .0001
Non	234,442(42.95)	3,738(53.95)	1,916(52.18)	2,817(52.61)	
Mild to moderate	264,341(48.43)	2,826(40.79)	1,539(41.91)	2,247(41.97)	
Heavy	47,041(8.62)	365(5.27)	217(5.91)	290(5.42)	
Regular exercise (n)^§^	122,611(22.46)	1,010(14.58)	476(12.96)	655(12.23)	< .0001
Low income (n)^||^	112,089(20.54)	1,570(22.66)	823(22.41)	1,222(22.82)	< .0001
Comorbidities					
DM (n)	51,701(9.47)	409(5.9)	205(5.58)	244(4.56)	< .0001
Hypertension (n)	157,011(28.77)	1,052(15.18)	490(13.34)	641(11.97)	< .0001
Dyslipidemia (n)	103,324(18.93)	620(8.95)	291(7.92)	352(6.57)	< .0001
CKD (n)	42,114(7.72)	378(5.46)	202(5.5)	315(5.88)	< .0001
Laboratory findings					
BMI (kg/m^2^)	24.1 ± 2.73	19.02 ± 1.46	18.32 ± 0.87	17.39 ± 0.77	< .0001
WC (cm)	82.01 ± 8.4	71.12 ± 6.13	69.42 ± 5.44	67.66 ± 5.4	< .0001
Systolic BP (mmHg)	123.86 ± 14.05	118.58 ± 14.3	118.03 ± 14.61	116.65 ± 14.15	< .0001
Diastolic BP (mmHg)	78.01 ± 9.64	74.53 ± 9.64	74.31 ± 9.73	73.6 ± 9.56	< .0001
Fasting glucose	99.01 ± 24.78	94.93 ± 23.96	94.56 ± 22.84	93.4 ± 22.22	< .0001
Total cholesterol (mg/dL)	198.87 ± 39.46	188.19 ± 44.31	187.09 ± 33.03	185.12 ± 37.72	< .0001
HDL	54.35 ± 28.43	61.08 ± 23.88	62.29 ± 29.08	63.15 ± 28.11	< .0001
LDL	118.8 ± 91.86	109.42 ± 107.8	107.79 ± 56.01	106.65 ± 63.76	< .0001
eGFR (ml/min/1.73m^2^)	82.97 ± 36.12	86.88 ± 29.59	88.44 ± 34.56	87.72 ± 29.99	< .0001
TG	123.72(123.53–123.91)	89.63(88.56–90.71)	88(86.59–89.42)	82.78(81.74–83.83)	< .0001

DM, diabetes mellitus; CKD, chronic kidney disease; BMI, body mass index; WC, waist circumference; BP, blood pressure; HDL, high-density lipoprotein; LDL, low-density lipoprotein; eGFR, estimated glomerular filtration rate; TG, triglyceride.

Numeric parameters are expressed as mean ± standard deviation and categorical parameters are expressed as counts and percentages in parentheses.

*Underweight was defined as body mass index under 18.5 kg/m^2^.

^†^Cumulative number of underweight diagnosed at each health examination (0–3 times).

^‡^Alcohol consumption was divided into 3 categories; Non (no alcohol consumption), Mild (under 30 g/day consumption), and heavy (over 30 g/day consumption).

^§^Regular exercise is defined as performing over 30 min moderate intensity exercise over 5 times per a week or over 20 min vigorous intensity exercise over 3 times per a week.

^||^Low income is defined as total household monthly income belongs to lower 20% group among Korean entire population.

### The incidence and risk of fractures according to body mass index

The IRs per 1000PY of newly diagnosed fracture were 10.41 (95%CI; 9.70 – 11.12), 8.62 (95%CI; 8.48 – 8.77), 7.91 (95%CI; 7.76 – 8.07), 7.62 (95%CI; 7.48 – 7.77), and 7.80 (95%CI; 7.29 – 8.31) who were underweight, normal, overweight, obesity, and severe obesity, respectively (Table 2). Adjusted Cox’s proportional hazards regression analyses were performed to calculate adjusted HRs (model 3) for newly diagnosed fractures by the BMI at the index health screening examination. Underweight was associated with a significantly higher risk despite the adjustment for several potentially confounding variables (adjusted HR; 1.173 [95%CI; 1.093–1.259]).

**Table 2:** The risk of fracture according to body mass index using Cox regression analysis.

Body mass index (kg/m^2^)	No. of fracture	IR*	95% CI	Unadjusted			Model 1			Model 2			Model 3		
				HR	95% CI	p-Value	HR	95% CI	p-Value	HR	95% CI	p-Value	HR	95% CI	p-Value
< 18.5	825	10.41	9.70–11.12	1.209	1.127–1.296		1.177	1.097–1.262		1.169	1.090–1.254		1.173	1.093–1.259	
≥ 18.5 and < 23	13,884	8.62	8.48–8.77	1		< 0.001	1		< 0.001	1		< 0.001	1		< 0.001
≥ 23 and < 25	10,118	7.91	7.76–8.07	0.918	0.894–0.941		0.945	0.921–0.969		0.947	0.923–0.972		0.944	0.920–0.969	
≥ 25 and < 30	10,863	7.62	7.48–7.77	0.884	0.862–0.907		0.932	0.909–0.956		0.933	0.910–0.957		0.927	0.903–0.951	
≥ 30	890	7.80	7.29–8.31	0.905	0.846–0.968		0.954	0.892–1.021		0.949	0.887–1.016		0.936	0.874–1.002	

No, number; IR, incidence rate; HR, hazard ratio; 95% CI, 95% confidence interval.

*Incidence rate is defined as incidence rate per 1,000 person-year.

Model 1 was adjusted by age, and sex.

Model 2 was adjusted by age, sex, and other environmental factors such as smoking status, alcohol consumption, regular exercise, low income.

Model 3 was fully adjusted by age, sex, other environmental factors (smoking status, alcohol consumption, regular exercise, low income), and comorbidities (diabetes, hypertension, dyslipidemia, chronic kidney disease).

### Incidence and risk of fractures according to the cumulative number of underweight

A total of 36,580 fractures were detected (6.5%). The IR of fractures was 10.54 (95%CI 9.68–11.40) in the once-diagnosed underweight group, 9.88 (95%CI 8.74–11.03) in the twice-diagnosed underweight group, and 10.50 (95%CI 9.52–11.48) in the thrice-diagnosed underweight group, with the overall IR being higher in the underweight group. In contrast, there was no statistically significant serial increase in the number of fractures as the number of underweight diagnoses. The HRs were still statistically significant after adjusting for many variables. Underweight individuals diagnosed once, twice, or thrice had an adjusted HR (Model 3) for fractures of 1.227 (95%CI 1.130–1.332), 1.174 (95%CI 1.045–1.319), and 1.255 (95%CI 1.143–1.379), respectively (Table 3).

**Table 3:** The risk of fracture according to the cumulative number of the presence of low body weight using Cox regression analysis.

Cumulative number of low body weight	No. of fracture	IR*	95% CI	Unadjusted			Model 1			Model 2			Model 3		
				HR	95% CI	p-Value	HR	95% CI	p-Value	HR	95% CI	p-Value	HR	95% CI	p-Value
0	35,277	8.05	7.69–8.14	1		< 0.0001	1		< 0.0001	1		< 0.0001	1		< 0.0001
1	576	10.54	9.68–11.40	1.311	1.207–1.423		1.227	1.130–1.333		1.223	1.126–1.328		1.227	1.130–1.332	
2	286	9.88	8.74–11.03	1.229	1.094–1.381		1.179	1.050–1.325		1.170	1.042–1.315		1.174	1.045–1.319	
3	441	10.50	9.52–11.48	1.307	1.190–1.435		1.260	1.147–1.384		1.250	1.138–1.373		1.255	1.143–1.379	

No, number; IR, incidence rate; HR, hazard ratio; 95% CI, 95% confidence interval.

*Incidence rate is defined as incidence rate per 1,000 person-year.

Model 1 was adjusted by age, and sex.

Model 2 was adjusted by age, sex, and other environmental factors such as smoking status, alcohol consumption, regular exercise, low income.

Model 3 was fully adjusted by age, sex, other environmental factors (smoking status, alcohol consumption, regular exercise, low income), and comorbidities (diabetes, hypertension, dyslipidemia, chronic kidney disease).

### Risk of fracture according to temporal trends in body mass index changes

The IR was 8.06 (95%CI 7.98–8.15) in the N-to-N group, 10.25 (95%CI 9.09–11.41) in the N-to-U group, 10.01 (95%CI; 8.89–11.12) in the U-to-N group, and 10.49 (95%CI; 9.64–11.35) in the U-to-U group. Participants in the U-to-U, N-to-U, U-to-N group had a substantially increased risk of fractures after multivariate adjustment (HR; 1.250 [95%CI; 1.146–1.363], 1.171 [95%CI; 1.045–1.312], and 1.203[95%CI; 1.075–1.346], respectively). Adults over 40 years of age who have ever been underweight, even once, had a higher adjusted HR, and even if their weight changes, being underweight increased the risk of fracture (Table 4).

**Table 4:** The risk of fracture according to temporal changes in body mass index changes using Cox regression analysis.

Underweight changes*	No. of fracture	IR^†^	95% CI	Unadjusted			Model 1			Model 2			Model 3		
				HR	95% CI	p-Value	HR	95% CI	p-Value	HR	95% CI	p-Value	HR	95% CI	p-Value
N to N	35,447	8.06	7.98–8.15	1		< 0.0001	1		< 0.0001	1		< 0.0001	1		< 0.0001
N to U	300	10.25	9.09–11.41	1.274	1.137–1.427		1.175	1.049–1.317		1.167	1.041–1.307		1.171	1.045–1.312	
U to N	308	10.01	8.89–11.12	1.242	1.110–1.390		1.202	1.075–1.345		1.200	1.073–1.343		1.203	1.075–1.346	
U to U	525	10.49	9.64–11.35	1.304	1.196–1.421		1.255	1.151–1.368		1.245	1.142–1.357		1.250	1.146–1.363	

No, number; IR, incidence rate; HR, hazard ratio; 95% CI, 95% confidence interval; N, non-low body weight (body mass index ≥ 18.5 kg/m^2^); L, low body weight (body mass index < 18.5 kg/m^2^).

*Temporal changes of underweight status (first to 3^rd^ health screening) are divided into four groups: non-underweight to non-underweight, non-underweight to underweight, underweight to non-underweight, and underweight to underweight.

^†^Incidence rate is defined as incidence rate per 1,000 person-year.

Model 1 was adjusted by age, and sex.

Model 2 was adjusted by age, sex, and other environmental factors such as smoking status, alcohol consumption, regular exercise, low income.

Model 3 was fully adjusted by age, sex, other environmental factors (smoking status, alcohol consumption, regular exercise, low income), and comorbidities (diabetes, hypertension, dyslipidemia, chronic kidney disease).

## Discussion

Based on our knowledge, this is the first large population-based cohort study to establish the risk of fractures related with the cumulative burden of underweight. This study determined that underweight status increases the risk of fractures in people over 40 years of age, and increasing cumulative number in underweight does not enhance the risk of further fracture.

Despite the fact that the mechanism by which underweight increases the incidence of fractures is unknown, this study discovered that underweight is a risk factor for increased fractures^27^. Hypothesized to cause osteoporosis, being underweight in humans is frequently related with malnutrition. Malnutrition leads to bone deterioration and osteoporosis^28,29^. In addition, a low BMI is strongly associated with sarcopenia development. Previous research has demonstrated that malnourished people are more susceptible to sarcopenia^30^. Sarcopenia diminishes physical strength and muscular performance, leading to injuries that increase the probability of fracture^31,32^. Therefore, a lower BMI correlates with decreased BMD levels and diminished muscle strength. However, because this was a population-based cohort study utilizing the ICD-10 diagnostic, procedure and radiographic codes, actual skeletal muscle index and BMD scores were not available. Although this study cannot definitively explain the association between low BMI, BMD, and skeletal muscle index, the vast population database confirmed that low BMI is associated with fractures.

After adjusting for a number of factors, the association between underweight and fractures was analyzed. Compared to individuals who never had underweight, those who had been underweight at least once had an increased risk of fracture. In other words, the risk of fracture remained to increase regardless of the duration of underweight or the status of underweight; however, the risk of fracture does not increase if an individual consistently maintains a weight above the normal range. Individuals who have shifted from underweight to normal weight or normal weight to underweight are considered to have a normal weight but close to being underweight. It is believed that these people had low bone density and diminished muscle function, which raises the risk for fractures. Even if body weight is regained to a non-underweight status, adults over the age of 40 who have been underweight may have a loss in bone density or muscular strength due to an increase in fat mass relative to muscle mass^33,34^. Thus, adults who have ever been underweight may be at a higher risk for fractures than adults with a normal or higher body weight.

This is the only study to our knowledge that used a national database to analyze the risk of fracture in the general underweight population over 40 years. All citizens were enrolled in the national health insurance system, which is a substantial quantity of data. Furthermore, the database is regularly updated; hence, it yields substantial results that may be applied to the general population.

This study has some limitations. First, the BMD T-scores could not be directly validated. Underweight had an effect on the BMD score; however the exact effect was unknown in this investigation. Second, determining the precise number of fractures was difficult. Unlike other fractures, vertebral fractures are often asymptomatic and are more likely to be underestimated than actual fractures. Third, this study utilized a national database from one nation's national health insurance services, making it difficult to adapt to multiple ethnic groups. Because fractures were identified using the fracture diagnostic code in this analysis, we were unable to validate that all fractures were appropriately diagnosed. The best way to confirm the suggested algorithm of diagnostic codes is through validation studies. In order to identify fractures, the same operational definitions established in previous studies were utilized in this study^4,23^. To diagnose fractures as precisely as possible, we excluded individuals with previous fractures and employed a one-year lag time period after underweight diagnosis. It is highly probable that the incidence rate of fractures was significantly underestimated due to the implementation of the most conservative methodology in this study. Finally, we tried to analyze as many factors as possible. While analyzing and adjusting for confounding factors is an important to increase the reliability of a study, no study is ideal and there is always a possibility of unmeasured or unanalyzed confounding factors. Therefore, while efforts were made to adjust for confounding factors in this study to obtain more accurate results, future research is needed for better understanding, and its limitations should be taken into account.

In conclusion, this study investigated whether being underweight is an important factor that increases the risk of fracture in the Korean population over 40-year-old individuals using a nationwide population-based cohort. Adults over the age of 40 who were underweight had an increased risk of fractures, even if they returned to normal weight.

## Supplementary Information

Supplementary Information.

## Data Availability

All data generated or analyzed during this study are included in this published article.
