# Supplementary material for: Underweight and risk of fractures in adults over 40 years using the nationwide claims database
Source: Sci Rep. 2023 May 17;13:8013. doi: 10.1038/s41598-023-34828-y (PMC10192329; doi:10.1038/s41598-023-34828-y)
Supplement: Supplementary file 1 — Supplementary Information. [file 41598_2023_34828_MOESM1_ESM.docx]

**Appendix 1**. Definitions of covariates and measurements.

| **Diagnosis** | **ICD-10-CM code and medication** | **Number of diagnosis** | **Diagnosis test or treatment** | **Combination*** |
| --- | --- | --- | --- | --- |
| **Inclusion/Exclusion** |  |  |  |  |
| Vertebral fracture | S22.0, S22.1 S32.0, S32.8 T08, M484 | Admission ≥ 1 or  Outpatient department with diagnostic test or treatment ≥ 1 | N0471, N0472, N0473,N 0474, N0630, G430, G440, G450, G460 |  |
| Hip fracture | S72.0, S72.1 |  | N0601, N0991, N0981, N0641, N0652, N0654, N0715, N0711, N0611, N2070, N2710 |  |
| Radius fracture | S52.5, S52.6 |  | N1601, N1611, N1603, N1613, N0996, N0998, N0983  T6020, T6030, T6151, T6152 |  |
| Humerus fracture | S42.2, S42.3 |  | N0602, N0612, N0992, N0982, N0986, N0722, N2711, N2716, T6010, T6110 |  |
| **Comorbidities based on the last health examination** | | |  |  |
| **Comorbidities** |  |  |  |  |
| Hypertension | I10-I13, I15; and minimum 1 prescription of anti-hypertensive drug (thiazide, loop diuretics, aldosterone antagonist, alpha-/beta-blocker, calcium-channel blocker, angiotensin-converting enzyme inhibitor, or angiotensin II receptor blocker) | Admission ≥ 1 or outpatient department ≥ 2 | Systolic/diastolic blood pressure ≥ 140/90 mmHg | 1+2 or 3 |
| DM | E11-E14; and minimum 1 prescription of anti-diabetic drugs (sulfonylureas, metformin, meglitinides, thiazolidinediones, dipeptidyl peptidase-4 inhibitors, α-glucosidase inhibitors, or insulin) | Admission ≥ 1 or outpatient department ≥ 2 | Fasting glucose level ≥ 126 mg/dL | 1+2 or 3 |
| Dyslipidemia | E78 | Admission ≥ 1 or outpatient department ≥ 1 | Total cholesterol ≥ 240 mg/dL | 1+2 or 3 |
| CKD | N/A | N/A | eGFR<60ml/min/1.73m^2^ | 3 |
| **Definitions of life style behavior based on the last health examination questionnaire** | | | | |
| **Alcohol consumption** |  |  |  |  |
| Mild to moderate drinker | Alcohol consumption > 0g to < 30g per day | |  |  |
| Heavy drinker | Alcohol consumption ≥ 30g per day | |  |  |
| **Regular exercise** |  |  |  |  |
|  | performing over 30 minutes moderate intensity exercise over 5 times per a week or over 20 minutes vigorous intensity exercise over 3 times per a week | | | |
| **Smoking** |  |  |  |  |
| Ex-smoker | Ex-smoker at the 1st examination and sustaining non-smoking till the 2nd examination | |  |  |
| Current smoker | Current smoker at the 2nd examination regardless of the smoking status at the 1st examination. | | | |
| **Information of income** | | | | |
| Low income | Income belongs to lower 20% among the entire Korean population and supported by the Medical Aid program | | |  |

Abbreviation: N/A, not applicable; DM, diabetes mellitus; CKD, chronic kidney disease.

* Combination: 1= ICD-10-CM code and medication; 2 = Number of diagnosis; and 3 = Diagnosis test or treatment
